# Tuberculous Aneurysm of the Thoracic Aorta: A Diagnostic and Therapeutic Challenge in the Modern Era

**DOI:** 10.3390/jcm15083104

**Published:** 2026-04-18

**Authors:** Sanja Šarac, Momir Šarac, Rade Milić, Biljana Lazović-Popović, Jelena Vuković

**Affiliations:** 1 Clinic for Pulmonology, Military Medical Academy, 11042 Belgrade, Serbia; rade.milic1975@gmail.com (R.M.); jelena.vukovic.cv@gmail.com (J.V.); 2 Faculty of Medicine of the Military Medical Academy, University of Defence, 11042 Belgrade, Serbia; dr.momirsarac@gmail.com; 3 Clinic for Vascular and Endovascular Surgery, Military Medical Academy, 11042 Belgrade, Serbia; 4 Department of Pulmonology, University Clinical Hospital Center “Zemun”, 11080 Belgrade, Serbia; lazovic.biljana@gmail.com

**Keywords:** tuberculosis, aortic aneurysm, hemoptysis, polymerase chain reaction, aortoesophageal fistula

## Abstract

**Introduction**: Tuberculous aneurysm of the thoracic aorta (TBAA) is an extremely rare but potentially fatal manifestation of tuberculosis (TB). Clinical presentation may include hemoptysis in the absence of parenchymal lung abnormalities. **Case report:** We presented a 62-year-old male with cough, chest pain, and minimal hemoptysis. Diagnostic evaluation confirmed an aneurysm of the descending thoracic aorta at a site previously treated with endovascular repair, with no imaging findings suggestive of pulmonary TB. Bronchoscopy revealed blood in the main bronchi without an identifiable endobronchial source. The diagnosis of TB was established by polymerase chain reaction (PCR) testing of bronchial aspirate obtained during bronchoscopy. Emergency surgical intervention was recommended because of an impending aortic rupture, but the patient declined surgery. Standard antituberculous therapy was initiated, and the patient subsequently developed drug-induced liver injury, prompting temporary cessation of treatment. The clinical course was later complicated by the development of an aortoesophageal fistula (AEF), with significant implications for prognosis. **Conclusions:** Early recognition of TBAA, along with a multidisciplinary approach that integrates advanced diagnostic modalities, timely vascular intervention, and carefully managed antituberculous therapy, is essential to reduce mortality and optimize treatment outcomes.

## 1. Introduction

Thoracic aortic aneurysm (TAA) is a clinically heterogeneous and potentially life-threatening condition associated with substantial mortality due to dissection or rupture, while often remaining clinically silent until advanced stages. Contemporary pooled estimates from population-based studies indicate an incidence of approximately 5 to 10 cases per 100,000 person years [1]. More recent longitudinal cohort data have demonstrated higher age and sex standardized incidence rates reaching 13.8 per 100,000 person years, suggesting an increasing burden of diagnosed disease in current clinical practice [2]. These trends likely reflect population aging, expanded use of high-resolution imaging, and improved survival among patients with cardiovascular comorbidities.

The pathogenesis of TAA is multifactorial and includes degenerative medial disease related to hypertension and atherosclerosis, heritable connective tissue disorders such as Marfan syndrome and Loeys Dietz syndrome, bicuspid aortic valve associated aortopathy, and less commonly inflammatory or infectious etiologies. Infectious aneurysms account for approximately 0.7–3% of all aortic aneurysms and are most frequently caused by *Staphylococcus* species, *Salmonella* species, and invasive fungal pathogens including *Candida*, *Aspergillus*, and *Mucor* species [3]. Tuberculous thoracic aortic aneurysm represents an exceptionally rare manifestation of vascular tuberculosis. Reliable global epidemiologic estimates are lacking, and the condition is documented predominantly in isolated case reports and small case series [4]. Aneurysm formation most commonly results from direct extension of adjacent tuberculous foci, such as mediastinal lymph nodes or paravertebral abscesses, into the aortic wall, although hematogenous dissemination may also occur. Early descriptions of tuberculous involvement of the aorta date back to the late 19th century. TBAA is reported more frequently in regions with a high prevalence of tuberculosis, particularly in South and Southeast Asia and sub-Saharan Africa, and the available evidence is derived largely from individual case reports and limited series [5,6]. Given the risk of rupture, fistula formation, and overwhelming infection, early recognition, rigorous risk stratification based on aneurysm diameter and growth kinetics, and timely integration of antimicrobial therapy with open or endovascular repair are essential to optimize clinical outcomes [7,8]. 

This case report was prepared in accordance with the CARE guidelines, and the CARE Checklist is provided as Supplementary Material.

## 2. Case Presentation

A 62-year-old man was admitted to our clinic with a two-month history of dry cough, chest pain, and minimal hemoptysis. On the day of admission, he expectorated approximately 10 mL of dark blood. His cardiovascular history was notable for thoracic endovascular aortic repair (TEVAR) and stenting of the left common iliac artery performed two years earlier. He also reported an episode of hemoptysis approximately one year prior, for which the etiology remained unconfirmed.

**Figure 1:**
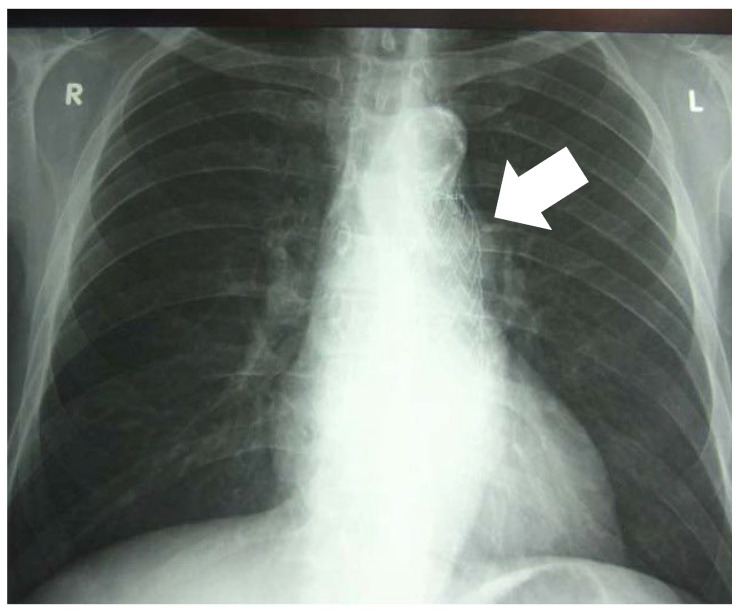
Chest radiography showed no pathological changes in the pulmonary parenchyma. Radiopaque material consistent with prior endovascular intervention was visible in the projection of the thoracic aorta (arrow).

On admission, chest radiography showed no parenchymal lung abnormalities (Figure 1).

The patient subsequently underwent pulmonary evaluation. Physical examination was unremarkable. Routine blood and urine tests were within reference ranges, except for an elevated C-reactive protein level of 15.6 mg/L (reference range, 0–10 mg/L).

Computed tomography (CT) of the chest confirmed the prior endovascular aortic repair without additional intrathoracic abnormalities. No abnormalities were identified in the lung parenchyma or intrathoracic vasculature.

Flexible bronchoscopy demonstrated small amounts of blood in both main bronchi. After suctioning, no endobronchial source of bleeding was identified.

No acid-fast bacilli were detected on direct smear of sputum or bronchial aspirate. No pathogenic bacteria or fungi were isolated from these samples. Specimens were obtained for further microbiological evaluation, including PCR testing for *Mycobacterium tuberculosis* (MTB).

**Figure 2:**
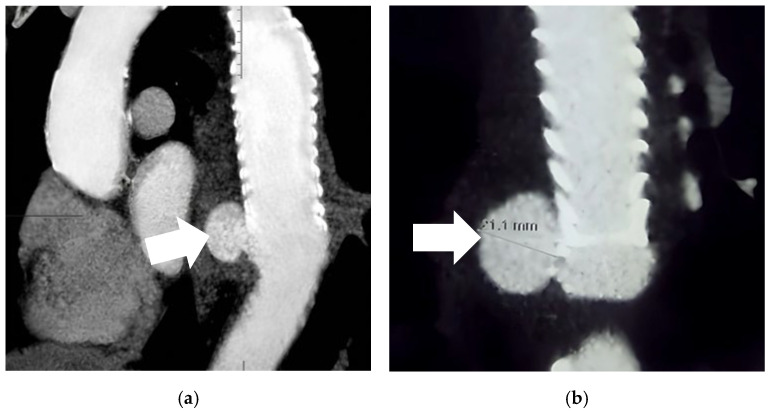
CTA demonstrating a 21 mm aneurysmal lesion of the descending thoracic aorta without contrast extravasation or endoleak (arrow) (**a**) with ×2 magnification and measurement indicated (**b**).

As no cause of hemoptysis was identified and given the patient’s prior thoracic aortic intervention, contrast-enhanced computed tomography angiography (CTA) of the chest was performed. CTA demonstrated a focal, well-defined dilation of the descending thoracic aorta at the distal landing zone of the previously implanted stent graft, measuring approximately 21 mm in diameter, with no mural thrombosis and no evidence of endoleak. The morphology of the lesion, including its localized appearance, relatively small size, and close relationship to the stent graft, favors a pseudoaneurysm or post-interventional change rather than a true aneurysm. No clear features of aortic dissection were identified, although subtle irregularity of the aortic wall at the graft interface could not be excluded. Mild periaortic soft tissue thickening was also noted, which may reflect an underlying inflammatory or infectious process (Figure 2).

On the same day, results from the previously obtained bronchial aspirate became available, and PCR testing was positive for MTB.

The patient was evaluated by a multidisciplinary team including a pulmonologist, radiologist, and vascular surgeon. The aortic lesion was considered most consistent with a TBAA. Given the substantial risk of rupture, urgent intervention was recommended. Open surgical reconstruction was the preferred definitive treatment, while repeat endovascular repair was reserved as a temporizing option in the setting of life-threatening hemorrhage. Despite comprehensive counseling, the patient declined any invasive treatment. Pharmacologic therapy was subsequently initiated according to the standard regimen, consisting of isoniazid, rifampin, pyrazinamide, and ethambutol.

Seven days after treatment initiation, the patient developed features consistent with drug-induced liver injury (DILI), presenting with jaundice, nausea, and vomiting. Biochemical blood analysis demonstrated marked hepatocellular enzyme elevation, with aspartate aminotransferase of 839 U/L (reference range, 0–37 U/L), alanine aminotransferase of 258 U/L (reference range, 10–49 U/L), and lactate dehydrogenase of 268 U/L (reference range, 120–246 U/L). Serologic testing was negative for hepatotropic viruses and human immunodeficiency virus.

As the patient’s overall condition was stable and there was no hemoptysis, antituberculous therapy was temporarily withheld, and supportive care with hepatoprotective agents was initiated until clinical recovery. Three days later, the patient developed massive hematemesis followed by hemorrhagic shock. Cardiopulmonary resuscitation measures were unsuccessful, and the patient died.

**Figure 3:**
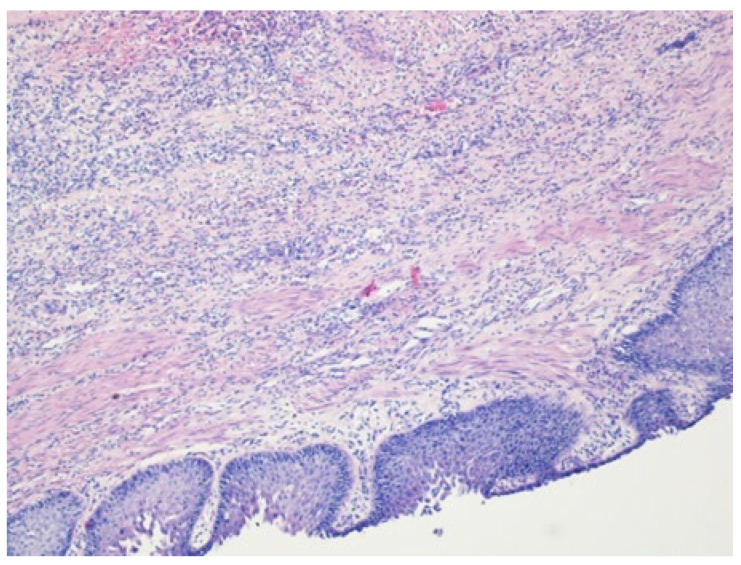
Histopathologic examination of the esophagus showing granulomatous infiltration (hematoxylin-eosin stain, ×40).

**Figure 4:**
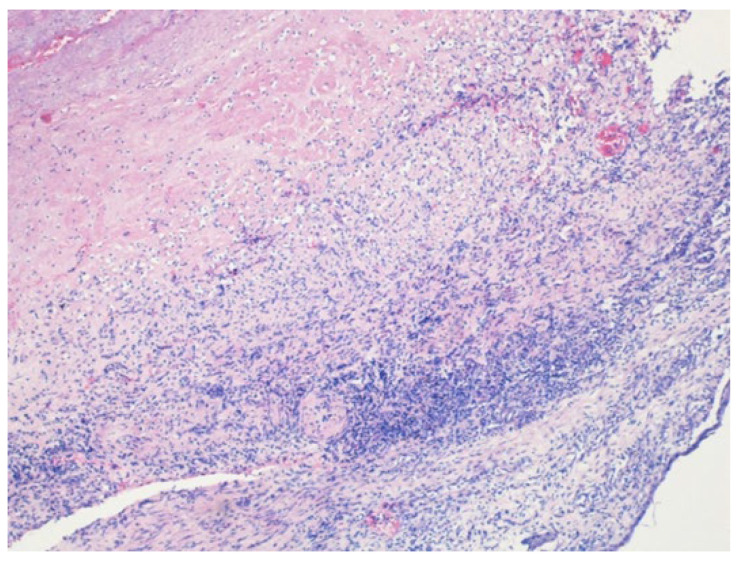
Histopathologic examination of the aortic aneurysm showing granulomatous infiltration (hematoxylin-eosin stain, ×40).

**Figure 5:**
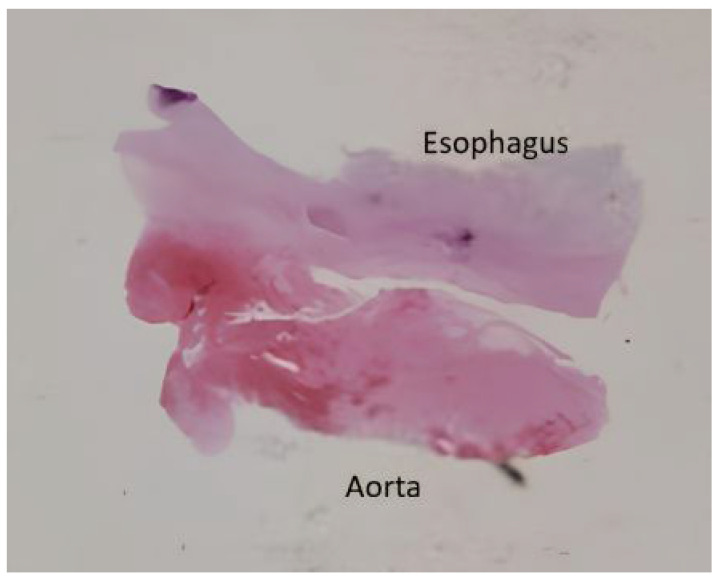
Histopathologic specimen of an aortoesophageal fistula.

Written informed consent for autopsy was obtained from the patient’s spouse in accordance with applicable ethical and institutional regulations. Clinical autopsy revealed foci of caseous necrosis in the upper lobe of right lung, an aneurysm of the descending aorta, an AEF, and hepatitis. The cause of death was massive hemorrhage due to the AEF. Histopathologic examination confirmed a ruptured AEF with an aneurysmal aortic pouch, marked inflammatory infiltrates, and multiple caseating epithelioid granulomas with multinucleated giant cells and associated lymphocytic infiltration, findings consistent with TB (Figure 3, Figure 4 and Figure 5).

**Figure 6:**
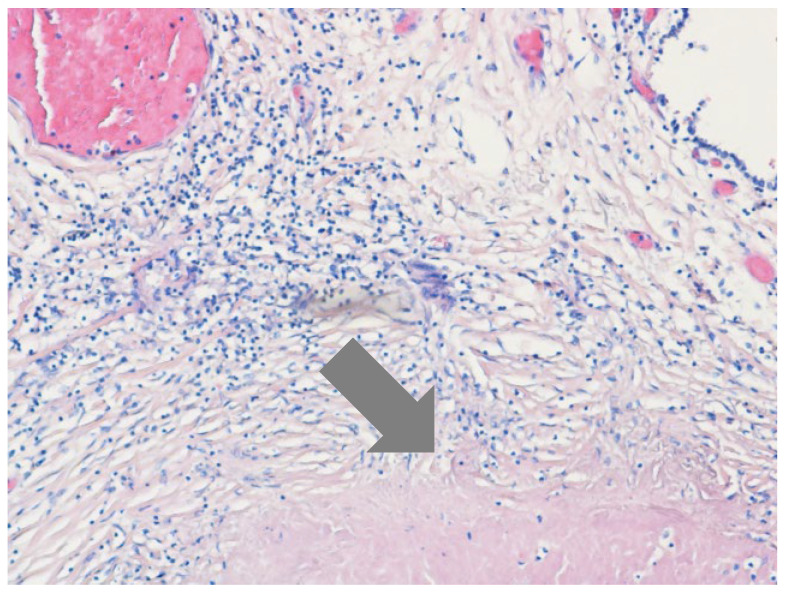
Histopathologic examination of the pulmonary parenchyma showing granulomatous infiltration with caseous necrosis (arrow) (hematoxylin-eosin stain, ×100).

Although no pathological changes were identified in the lung parenchyma on CT, a tuberculous lesion with caseous necrosis was identified in the upper lobe of the right lung (Figure 6).

Five weeks after the patient’s death, results of Löwenstein-Jensen cultures became available. MTB was isolated and was susceptible to all first-line antituberculous drugs, indicating that the initiated therapy was appropriate.

The clinical course is summarized in Table 1.

## 3. Discussion

TBAA is a rare but highly lethal manifestation of extrapulmonary tuberculosis, most commonly resulting from contiguous spread of infection or, less frequently, hematogenous dissemination [9]. Despite its rarity, it carries substantial clinical significance, as symptoms such as hemoptysis, chest pain, or sentinel bleeding may precede catastrophic rupture [10]. This pattern was observed in the present case, in which a sentinel bleed was followed by fatal hemorrhage due to an AEF. Such presentations pose a substantial diagnostic and therapeutic challenge in both endemic and nonendemic settings and require a high index of suspicion, particularly in the absence of pulmonary abnormalities and in patients with vascular risk factors or a history of prior aortic intervention. TBAA frequently presents as a pseudoaneurysm, characterized by localized arterial dilatation without preservation of normal vessel wall architecture, which is associated with a high risk of rupture [11].

Advanced imaging plays a central role in diagnostic refinement and follow-up. CTA remains the primary modality for defining aneurysm morphology and detecting complications, although interpretation may be limited by stent-graft artifacts or nonspecific mural changes. In selected cases, fluorine-18 fluorodeoxyglucose positron emission tomography/computed tomography may aid in differentiating infectious from noninfectious processes. This multimodal approach is particularly relevant in patients with prior aortic interventions, in whom differentiation between infection, graft-related complications, and sterile inflammation has direct therapeutic implications [12].

In infective aortic aneurysms, early multidisciplinary evaluation and a combined therapeutic strategy are essential. Definitive management typically requires anatomical exclusion of the aneurysm through open surgical reconstruction or, in selected cases, endovascular techniques, followed by prolonged pathogen-directed antimicrobial therapy. Outcomes are consistently superior when source control is achieved compared with medical therapy alone [4].

In cases complicated by AEF, management is particularly challenging due to the immediate risk of massive, life-threatening hemorrhage requiring urgent control. Open surgical repair enables radical debridement of infected tissue but carries substantial perioperative risk, especially in hemodynamically unstable patients. In contrast, TEVAR provides rapid hemorrhage control and is increasingly used as a life-saving bridging strategy, although it does not eradicate infection and should not be considered definitive therapy in most cases [13].

Only a limited number of cases of tuberculous thoracic aortic aneurysm or pseudoaneurysm complicated by AEF have been reported, typically in younger patients and associated with high mortality despite treatment (Table 2).

In the present case, the absence of pulmonary abnormalities on chest radiography and CT redirected diagnostic attention toward the great vessels. CTA identified the aortic lesion, while PCR positivity for MTB in bronchial aspirate supported an infectious etiology. Microbiological confirmation was essential for guiding management [22].

Although PCR positivity suggested a tuberculous etiology, alternative causes of the aneurysm required careful consideration, including non-tuberculous etiologies and graft-related infection following prior TEVAR. The known limitations of PCR testing, including the potential for false-positive results or contamination, also required cautious interpretation [23]. The working diagnosis was established through an integrated assessment of clinical, radiological, and microbiological findings, with definitive confirmation obtained postmortem. Another important consideration is that the underlying aortic pathology may have been present but unrecognized at the time of the initial TEVAR, raising the possibility of early or subclinical infectious involvement contributing to subsequent vascular wall instability.

The development of AEF was most likely driven by progressive infectious destruction of the aortic wall in the setting of tuberculous aortitis, followed by erosion into the adjacent esophagus. Imaging demonstrated focal aneurysmal or pseudoaneurysmal dilation at the site of prior endovascular repair, without overt signs of fistulization, but with surrounding inflammatory changes that extended due to the spread of infection and led to fistula formation. This mechanism was confirmed at autopsy. The patient declined the proposed surgical intervention, precluding implementation of the recommended combined treatment strategy and significantly influencing the clinical course and outcome.

Management was further complicated by DILI, which necessitated interruption of standard antituberculous therapy. Severe hepatocellular injury required complete discontinuation of treatment. Clinical guidelines recommend withholding hepatotoxic agents when alanine aminotransferase or aspartate aminotransferase levels reach ≥5 times the upper limit of normal in asymptomatic patients or ≥3 times in symptomatic patients, with consideration of bridging using non-hepatotoxic regimens when treatment cannot be deferred. Alternative regimens including ethambutol, fluoroquinolones, or aminoglycosides may be considered to maintain antimicrobial coverage when clinically feasible. In this patient, however, the severity of hepatocellular injury and the absence of ongoing hemoptysis supported a cautious approach with complete interruption of therapy and close monitoring until biochemical recovery. Less hepatotoxic regimens were not initiated due to the severity of liver injury and the temporarily stable clinical condition, which may have contributed to insufficient infection control in a high-risk vascular focus.

After normalization of liver enzymes, therapy is typically reintroduced in a stepwise manner, with rifampicin first, followed by isoniazid, while pyrazinamide is added last or omitted if hepatotoxicity recurs, with at least weekly monitoring of liver biochemistry [24]. This strategy may be difficult to implement in severe extrapulmonary tuberculosis involving critical vascular structures, where sustained antimicrobial pressure is essential. The present case highlights the challenge of balancing treatment-related toxicity with the need for uninterrupted therapy.

In the present case, the relatively small diameter of the aortic lesion (21 mm) and its location at the distal landing zone of the previously implanted stent graft support the diagnosis of a pseudoaneurysm rather than a true aneurysm. This interpretation is consistent with the known risk of intimal injury and localized wall disruption following endovascular procedures. In the presence of confirmed MTB infection, superimposed tuberculous aortitis likely contributed to structural weakening and progression of the lesion. These findings reflect a complex interplay between post-interventional vascular injury and infection, ultimately leading to pseudoaneurysm formation and fistulization.

Prognosis in TBAA is primarily determined by the risk of rupture and the development of AEF, a complication associated with extremely high mortality. Management requires prompt recognition, rapid hemorrhage control, eradication of infection, and close follow-up [25].

This case integrates several high-risk and interrelated factors, including prior TEVAR as a substrate for vascular wall injury, superimposed tuberculous infection, and early development of DILI that limited therapeutic options. The absence of pulmonary imaging findings despite confirmed tuberculosis further complicated the diagnostic process and delayed recognition of the vascular source of symptoms. Together, these elements illustrate a clinically relevant but underrecognized scenario in which post-interventional vascular injury and infection act synergistically, leading to rapid disease progression and the development of a fatal AEF.

Overall, this case highlights three key clinical considerations. First, TBAA should be considered in the differential diagnosis of hemoptysis even in the absence of pulmonary parenchymal abnormalities. Second, microbiological confirmation is essential for guiding therapeutic decision-making. Third, optimal management requires timely surgical or endovascular source control combined with antituberculous therapy within a multidisciplinary framework [26].

## 4. Conclusions

Tuberculous aneurysm of the thoracic aorta should be considered in the differential diagnosis of hemoptysis, particularly in patients with prior aortic intervention, even in the absence of pulmonary abnormalities. Management is complex and may be further constrained by treatment-related toxicity, while the risk of catastrophic rupture, as illustrated by AEF in the present case, underscores the need for early recognition and timely intervention.

## Figures and Tables

**Table 1:** Clinical timeline of the patient.

Time Point	Event
2 years before admission	Thoracic endovascular aortic repair (TEVAR)
1 year before admission	Episode of hemoptysis of unknown etiology
2 months before admission	Onset of dry cough and chest pain
Day of admission	Hemoptysis (<10 mL) Chest radiography without abnormalities
During hospitalization Day 2–4	CT of the chest confirmed the prior endovascular aortic repair without additional intrathoracic pathology Bronchoscopy showed blood without endobronchial source of bleeding
Day 5	CTA revealed a lesion of the descending thoracic aorta without pulmonary abnormalities Positive PCR result for *Mycobacterium tuberculosis* from bronchial aspirate
Day 6	Multidisciplinary evaluation Urgent surgical intervention recommended but declined by the patient
Day 7	Initiation of antituberculous therapy
Day 14	Confirmation of drug-induced liver injury
Day 15	Discontinuation of therapy
Day 18 Outcome	Massive hematemesis followed by hemorrhagic shock Death despite resuscitation efforts
5 weeks after death	AEF and tuberculous aortitis confirmed

**Table 2:** Summary of reported cases of tuberculous aneurysm and pseudoaneurysm of the thoracic aorta complicated by AEF.

Author Year	Age/Sex	Clinical Presentation	Diagnostic Procedures	Management	Outcome
Hong et al. 1965 [14]	28 female	Cough, fever, chest pain, anorexia	Chest radiography; Mantoux test; autopsy with histopathology	antituberculous therapy	died
Robbs JV et al. 1976 [15]	49 female	melena hematemesis	chest x-ray, esophagogastroscopy	Open surgical repair	died
Catinella FP et al. 1988 [16]	87 female	Pulmonary tuberculosis, dysphagia	Chest radiography; CT; barium esophagogram; esophagoscopy	antituberculous therapy	died
Amonkar GP et al. 2009 [17]	60 male	massive hematemesis	autopsy	-	died
Sato T et al. 2015 [18]	50 female	hematemesis	Chest radiography; CT; esophagoscopy	TEVAR + antituberculous therapy	survived
Na JY et al. 2015 [19]	68 male	dyspepsia, vomiting massive hemoptysis	autopsy	-	died
Vijayvergiya R et al. 2020 [20]	42 female	Miliary tuberculosis, dysphagia, hematemesis, cachexia	CT; CTA; barium esophagogram; esophagoscopy	TEVAR + antituberculous therapy + antibiotics	died (7 months after TEVAR)
Hafezeftekhari S et al. 2023 [21]	36 female	Nausea, vomiting	Chest radiography; CT; esophagoscopy	supportive treatment	died

## Data Availability

The original data presented in this study are included in this article. Further inquiries may be directed to the corresponding author.

## Supplementary Materials

The following supporting information can be downloaded at: https://www.mdpi.com/article/10.3390/jcm15083104/s1, Table S1: CARE Checklist

## Abbreviations

The following abbreviations are used in this manuscript:

TAA	Thoracic Aortic Aneurysm
TB	Tuberculosis
MTB	*Mycobacterium tuberculosis*
TBAA	Tuberculous Aortic Aneurysm
TEVAR	Thoracic Endovascular Aortic Repair
CT	Computed Tomography
CTA	Computed Tomography Angiography
PCR	Polymerase Chain Reaction
AEF	Aorto-Esophageal Fistula
DILI	Drug-Induced Liver Injury
