# Supplementary material for: Tuberculous Aneurysm of the Thoracic Aorta: A Diagnostic and Therapeutic Challenge in the Modern Era
Source: J Clin Med. 2026 Apr 18;15(8):3104. doi: 10.3390/jcm15083104 (PMC13117258; doi:10.3390/jcm15083104)
Supplement: Supplementary file 1 [file jcm-15-03104-s001.zip › jcm-4222256-supplementary.pdf]

CARE case report guidelines

CARE Checklist of information to include when writing a case report

(cc) BY-NC-ND

Topic	Item	Checklist item description	Reported on Line
**Title**	1	The diagnosis or intervention of primary focus followed by the words “case report”	Title page
**Key Words**	2	2 to 5 key words that identify diagnoses or interventions in this case report, including "case report"	Keywords section
**Abstract (no references)**	3a	Introduction: What is unique about this case and what does it add to the scientific literature?	Abstract
	3b	Main symptoms and/or important clinical findings	Abstract
	3c	The main diagnoses, therapeutic interventions, and outcomes	Abstract
	3d	Conclusion—What is the main “take-away” lesson(s) from this case?	Abstract
**Introduction**	4	One or two paragraphs summarizing why this case is unique (**may include references**)	Introduction section
**Patient Information**	5a	De-identified patient specific information.	Case presentation section
	5b	Primary concerns and symptoms of the patient.	Case presentation section
	5c	Medical, family, and psycho-social history including relevant genetic information	Case presentation section
	5d	Relevant past interventions with outcomes	Case presentation section
**Clinical Findings**	6	Describe significant physical examination (PE) and important clinical findings.	Case presentation section
**Timeline**	7	Historical and current information from this episode of care organized as a timeline	Table 1(Clinical Timeline)
**Diagnostic Assessment**	8a	Diagnostic testing (such as PE, laboratory testing, imaging, surveys).	Case presentation section
	8b	Diagnostic challenges (such as access to testing, financial, or cultural)	Case presentation section
	8c	Diagnosis (including other diagnoses considered)	Case presentation section
	8d	Prognosis (such as staging in oncology) where applicable	Discussion section
**Therapeutic Intervention**	9a	Types of therapeutic intervention (such as pharmacologic, surgical, preventive, self-care)	Case presentation section
	9b	Administration of therapeutic intervention (such as dosage, strength, duration)	Case presentation section
	9c	Changes in therapeutic intervention (with rationale)	Case presentation section
**Follow-up and Outcomes**	10a	Clinician and patient-assessed outcomes (if available)	Case presentation section
	10b	Important follow-up diagnostic and other test results	Case presentation section
	10c	Intervention adherence and tolerability (How was this assessed?)	Case presentation section
	10d	Adverse and unanticipated events	Case presentation section
**Discussion**	11a	A scientific discussion of the strengths AND limitations associated with this case report	Discussion section
	11b	Discussion of the relevant medical literature **with references**.	Discussion section
	11c	The scientific rationale for any conclusions (including assessment of possible causes)	Discussion section
	11d	The primary “take-away” lessons of this case report (without references) in a one paragraph conclusion	Conclusion
**Patient Perspective**	12	The patient should share their perspective in one to two paragraphs on the treatment(s) they received	Not applicable
**Informed Consent**	13	Did the patient give informed consent? Please provide if requested	**Yes** ☒ **No** ☐
